# A case of mixed adenoneuroendocrine carcinoma (MANEC) arising in Barrett’s esophagus: literature and review

**DOI:** 10.1186/s40792-018-0454-z

**Published:** 2018-05-08

**Authors:** Tetsuro Kawazoe, Hiroshi Saeki, Keitaro Edahiro, Shotaro Korehisa, Daisuke Taniguchi, Kensuke Kudou, Ryota Nakanishi, Nobuhide Kubo, Koji Ando, Yuichiro Nakashima, Eiji Oki, Minako Fujiwara, Yoshinao Oda, Yoshihiko Maehara

**Affiliations:** 1 Department of Surgery and Science, Graduate School of Medical Sciences, Kyushu University, 3-1-1 Maidashi, Higashi-ku, Fukuoka, 812-8582 Japan; 2 Department of Anatomic Pathology, Pathological Sciences, Graduate School of Medical Sciences, Kyushu University, Fukuoka, Japan

**Keywords:** Mixed adenoneuroendocrine carcinoma (MANEC), Barrett’s esophagus, Neuroendocrine tumor

## Abstract

**Background:**

Mixed adenoneuroendocrine carcinoma (MANEC) is defined as a neoplasm composed of both exocrine and endocrine carcinomas, each comprising at least 30% of the tumor. MANEC can occur in various organs of the gastrointestinal tract, including the esophagus, stomach, and colon. We herein provide the first case report of surgically resected MANEC arising in Barrett’s esophagus (BE).

**Case presentation:**

A 70-year-old man presenting with abdominal pain was referred to our hospital. Upper endoscopy showed a type 0-IIa + IIc elevated lesion adjacent to BE. According to a biopsy specimen, the elevated lesion was diagnosed as adenocarcinoma with neuroendocrine differentiation. No lymphatic or distant metastasis was detected in the preoperative examination. Laparoscopic distal esophagectomy and proximal gastrectomy were performed, and a diagnosis of MANEC in BE was determined according to the surgically resected specimen.

**Conclusions:**

A very rare case of MANEC in BE was detected. BE can be the origin of esophageal MANEC as well as adenocarcinoma. Due to the small number of esophageal or esophagogastric MANEC cases reported, further accumulation of such cases is necessary to recommend an optimal management strategy for esophageal or esophagogastric MANEC.

## Background

Since Oberndorfer first reported a case of “karzinoide” in 1907, carcinoid tumors or neuroendocrine tumors (NETs) have been debated, and the treatment strategy for these tumors has been a contentious subject [1]. In the 2010 World Health Organization (WHO) classification of neuroendocrine neoplasms in the digestive system, mixed adenoneuroendocrine carcinoma (MANEC) is defined as a neoplasm composed of both exocrine and endocrine carcinomas, each comprising at least 30% of the tumor [2]. MANECs in the gastrointestinal tract can occur in various organs such as the esophagus [3], stomach [4, 5], and colon [6].

Barrett’s esophagus (BE) is an esophageal lesion exhibiting Barrett’s mucosa, which is a columnar epithelial metaplasia that extends from the stomach to the esophagus, as confirmed by endoscopy. Histologically, Barrett’s mucosa is characterized by the following three features: proper esophageal glands or ducts beneath the overlying columnar epithelium, squamous epithelial islets in the columnar epithelium, and double-layered lamina muscularis mucosae [7]. The malignant progression of BE to adenocarcinoma has been confirmed [8], and the incidence of esophageal adenocarcinoma has increased recently, especially in Western countries [9]. To the best of our knowledge, there has been only one brief case report of MANEC developing in BE, which was resected by endoscopic submucosal dissection [10].

The incidence of esophageal carcinoma in Japan is 31.0/100,000 person-years according to the National Cancer Center, Japan [11]. Among esophageal carcinomas, Barrett’s adenocarcinoma and endocrine cell carcinoma are extremely rare, with rates of 1.1 and 0.2%, respectively, among 5878 total esophageal cancer cases, according to the Comprehensive Registry of Esophageal Cancer in Japan, 2010 [12].

In this report, we present a case of MANEC that arose in histologically confirmed BE, along with a brief literature review. We herein provide the first report of surgically resected MANEC in BE.

## Case presentation

**Fig. 1:**
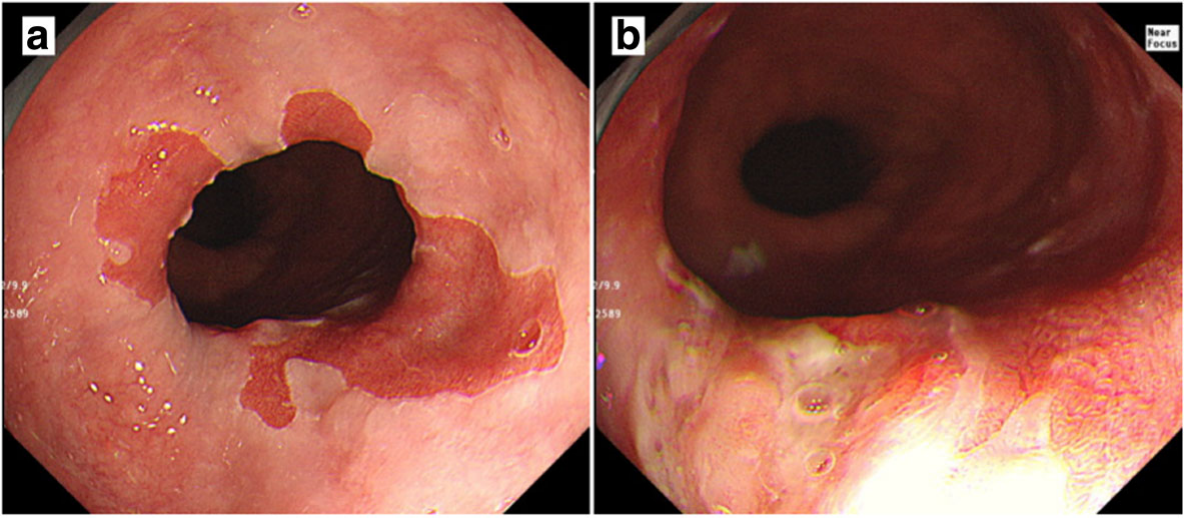
Upper endoscopy. Upper endoscopy showed an elongated columnar epithelium from the squamocolumnar junction indicating BE (**a**) and a type 0-IIa + IIc elevated lesion adjacent to the BE lesion (**b**)

**Fig. 2:**
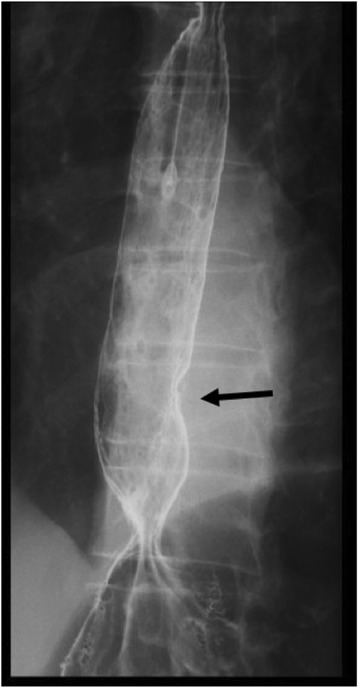
Esophagography. Esophagography showed a type 0-IIa + IIc elevated lesion (15 mm in size) on the left wall of the lower esophagus, and the tumor exhibited arcuate change suggesting submucosal invasion

A 70-year-old man presenting with abdominal pain was referred to our hospital for further examination. He had no past history without appendicitis operated during his childhood. He claimed no family history of cancer or any genetic disorders. Laboratory data revealed no significant abnormalities. Serum levels of tumor markers such as carbohydrate antigen 19-9 (25.7 U/ml, normal range < 37 U/ml) and neuron-specific enolase (9.4 ng/ml, normal range < 16.3 ng/ml) were within the normal ranges, while the carcinoembryonic antigen level was slightly increased (5.6 ng/ml, normal range < 5.0 ng/ml). The patient underwent upper endoscopy, which revealed BE and a type 0-IIa + IIc elevated lesion adjacent to the BE lesion (Fig. 1). The tumor biopsy specimen confirmed well to poorly differentiated adenocarcinoma with neuroendocrine differentiation. Esophagography showed a type 0-IIa + IIc elevated lesion in the left wall of the lower esophagus, with a size of 15 mm and an estimated depth invading into the submucosa (Fig. 2). Thoracic and abdominal contrast-enhanced computed tomography revealed no evidence of an esophageal mass, lymphatic metastasis, or distant metastasis.

The patient was clinically diagnosed with adenocarcinoma developing in BE. He subsequently underwent laparoscopic distal esophagectomy, proximal gastrectomy, regional lymph node dissection, and double-tract reconstruction with curative intent. Intraoperative findings revealed no tumor invasion into the esophageal adventitia.

**Fig. 3:**
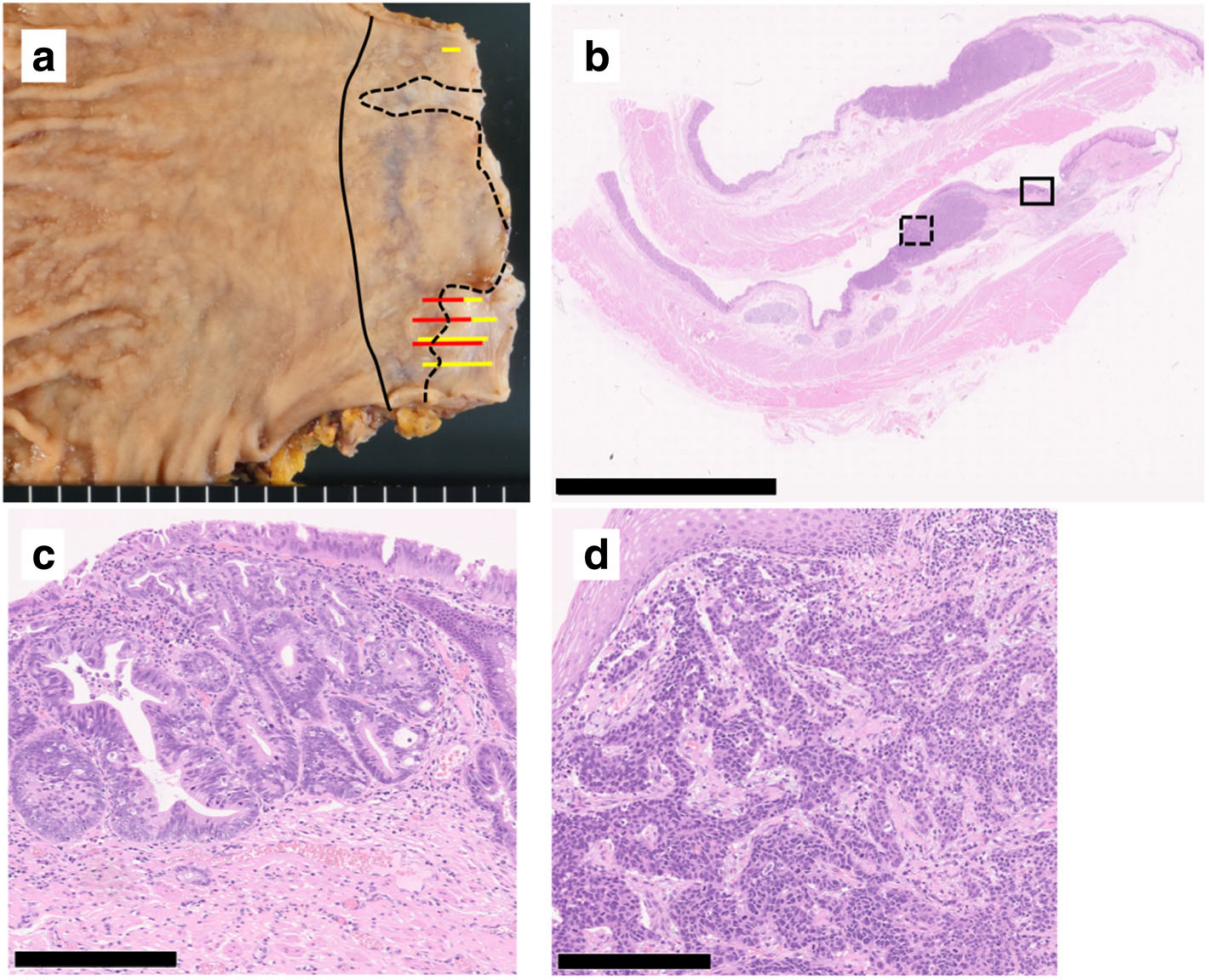
Histological findings of MANEC. Macroscopically, a type 0-IIa + IIc lesion measuring 25 × 10 mm was detected in the esophagogastric junction. The black solid line indicates the esophagogastric junction, the black dotted line the squamocolumnar junction, the yellow line the adenocarcinoma component, and the red line the NEC component (**a**). A loupe image of the lesion is shown (**b**, scale bar 10 nm). The solid rectangle indicates well differentiated adenocarcinoma (**c** scale bar 250 μm), and the dotted rectangle indicates an area of proliferation of round-shaped carcinoma cells with hyperchromatic nuclei and scant cytoplasm in a nested pattern, indicative of small cell NEC (**d** scale bar 250 μm)

**Fig. 4:**
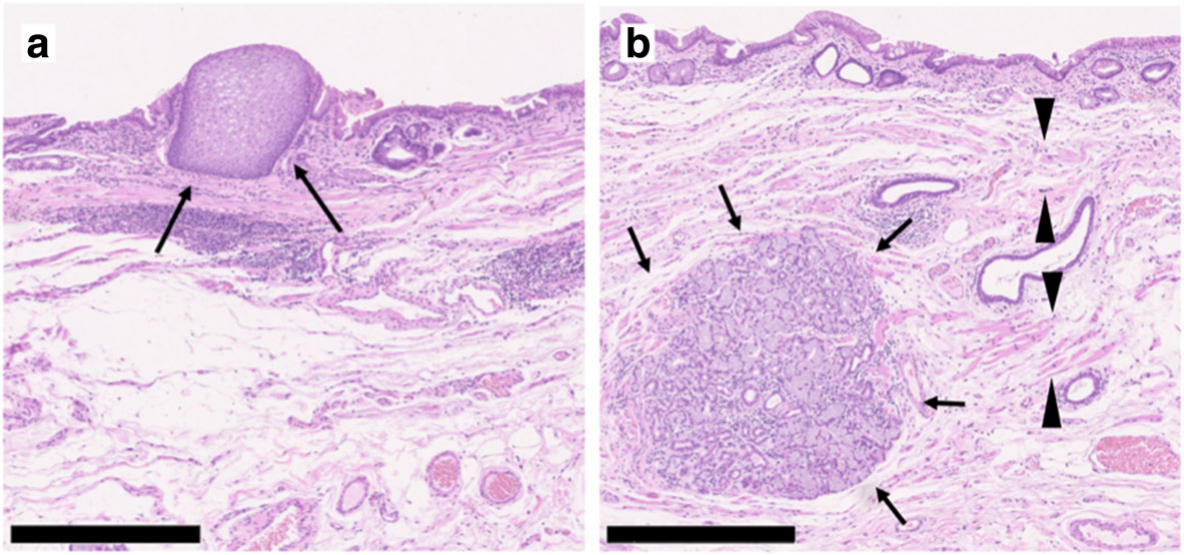
Histological findings of Barrett’s esophagus. BE was recognized histologically. Some islands of squamous epithelium (**a**, arrow), esophageal glands beneath the columnar epithelium (**b**, arrow), and double-layered muscularis mucosae (**b**, arrow head) were observed. All scale bars—500 μm

**Fig. 5:**
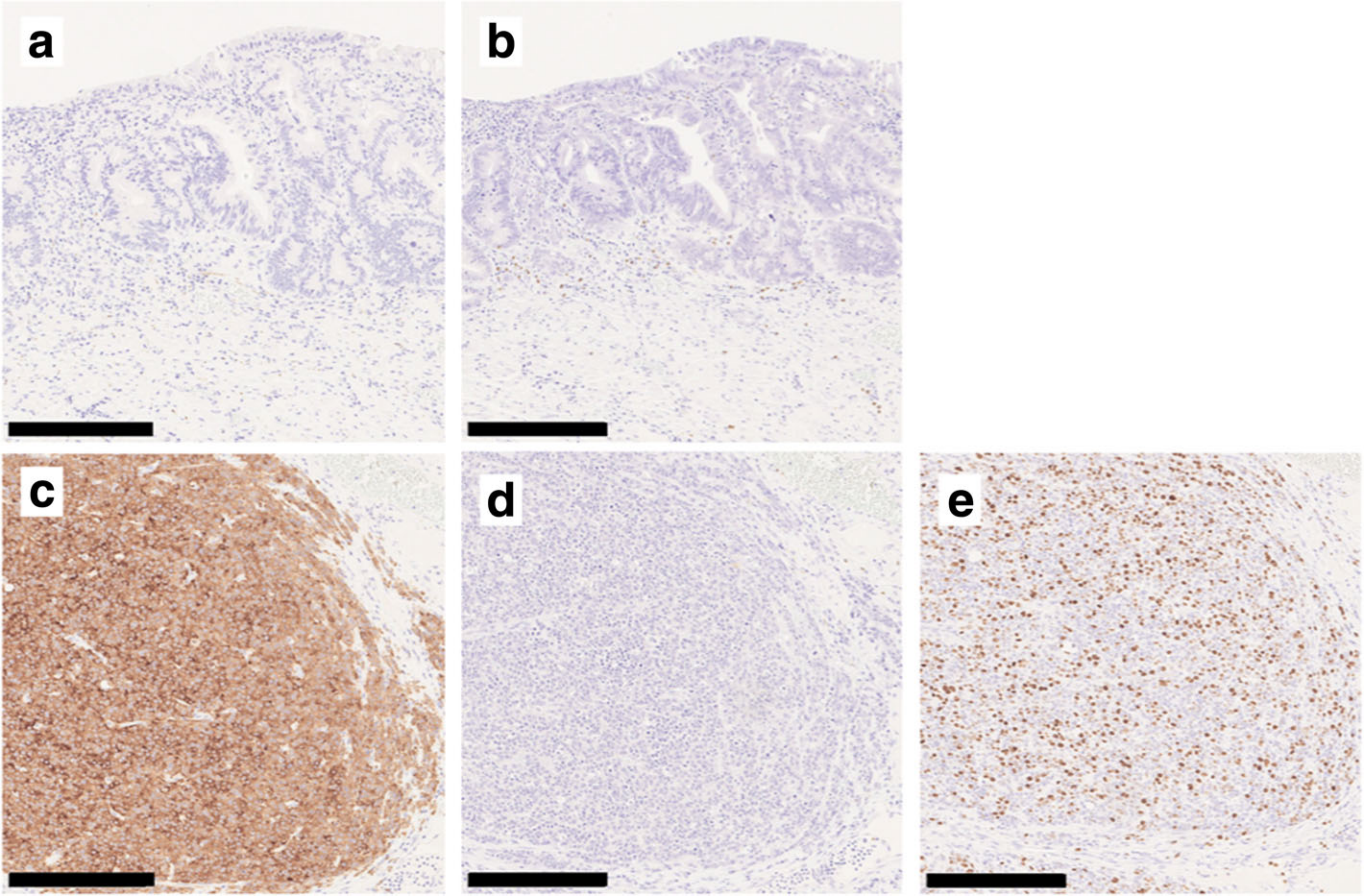
Immunohistochemical findings. Immunohistochemically, adenocarcinoma cells were negative for synaptophysin (**a**) and chromogranin A (**b**), while the round-shaped carcinoma cells were diffusely positive for synaptophysin (**c**), but negative for chromogranin A (**d**). The Ki67 labeling index was 50% (**e**). All scale bars—250 μm

In the resected specimen, a type 0-IIa + IIc lesion measuring 25 × 10 mm was macroscopically observed in the esophagogastric junction. Histological examination revealed that the neoplastic lesion was composed of well to moderately differentiated adenocarcinoma invading the submucosa and an area of proliferation of round-shaped carcinoma cells in a nested or sheeted pattern (Fig. 3). The round-shaped carcinoma cells contained hyperchromatic small nuclei with scant cytoplasm indicating small cell NEC [13]. Histological examination also revealed some islands of squamous epithelium, esophageal glands beneath the columnar epithelium, and double-layered muscularis mucosae, indicating BE near the neoplastic lesion (Fig. 4). Immunohistochemically, the adenocarcinoma cells were negative for chromogranin A and synaptophysin, whereas the round-shaped carcinoma cells were diffusely positive for synaptophysin but negative for chromogranin A and p40. The Ki67 labeling index was 50% (Fig. 5). Based on these findings, a diagnosis of MANEC arising in BE was made. No metastasis of the carcinoma cells into resected regional lymph nodes was detected. The final pathological stage was T1 N0 M0 stage I according to the seventh edition of the Union for International Cancer Control classification.

The postoperative course was uneventful. The patient was discharged on postoperative day 19 and has been followed carefully without any adjuvant chemotherapy and no evidence of tumor recurrence 4 months after surgical resection.

## Discussion

The 2010 WHO classification categorizes NETs into grade 1 and 2 NETs, neuroendocrine carcinoma (NEC), MANEC, and hyperplastic and preneoplastic lesion [2]. In this classification, grade 1 NETs (mitotic count < 2 per 10 high-power fields [HPFs]), grade 2 NETs (mitotic count 2–20 per 10 HPFs and/or Ki67 index > 20%), NEC (mitotic count > 20 per 10 HPFs and/or Ki67 index > 20%), and MANEC is defined as neoplasms comprised of both adenocarcinoma and NEC components in at least 30% of the tumor [2]. The tumor architecture is the most important diagnostic feature for MANEC, which is then confirmed by immunohistochemical findings such as chromogranin A, synaptophysin, CD56, and neuron-specific enolase expression [14]. Chromogranin A and synaptophysin expression is seen in 69.1 and 90.2%, respectively, of gastroenteropancreatic neuroendocrine neoplasms [15]. In the present case, almost equal proportions of the two components (adenocarcinoma and NEC) were confirmed. Immunohistochemically, the NEC component was positive for synaptophysin but negative for chromogranin A and p40. The Ki67 index was 50%.

La Rosa et al. categorized mixed exocrine–neuroendocrine neoplasms of the gastrointestinal tract into three subgroups: high-grade malignant neoplasms composed of mixed adenoma/adenocarcinoma and NEC, intermediate-grade malignant neoplasms composed of adenocarcinoma and grade 1/2 NET or amphicrine carcinoma, and mixed adenoneuroendocrine tumor composed of adenoma and NET [16]. According to this classification [16], our case fit the criteria of high-grade malignant MANEC.

BE is a precancerous condition in the development of esophageal adenocarcinoma, and it increases the risk of cancer by 11-fold [17]. Barrett’s mucosa exhibits the three pathological characteristics mentioned above [7]. In this case, some islands of squamous epithelium, esophageal glands beneath the columnar epithelium, and double-layered muscularis mucosae were confirmed histologically. Based on these features, MANEC arising in BE was diagnosed.

In the literature published to date, eight MANEC cases, including our case of MANEC located in the esophagus or esophagogastric junction, have been reported [3, 10, 18–21]. The clinicopathological features of the eight cases are shown in Table 1. Seven patients were male, and one patient was female. The mean age of the patients was 64 years (range, 57–70 years). The mean size of the tumors was 5.0 cm (range, 1.7–9.7 cm). Four cases developed in the esophagogastric junction and the other four in the esophagus. Seven cases were resected surgically, and one case with limited invasion to the submucosa was treated by endoscopic submucosal dissection. Lymph node metastases were observed in five cases. MANEC in BE was reported in only one case [10].

**Table 1:** Previously reported cases of esophageal or esophagogastric MANEC

Case	Age	Sex	Location	Treatment	Tumor size (cm)	Tumor depth	LN metastasis	Adjuvant chemotherapy	Recurrence	Prognosis	Author, year
1	64	Male	Ut	Surgery	1.7	T1	N0	None	None	Alive (16 months)	Kitajima, 2013
2	63	Male	EGJ	Surgery	9.7	T3	N2	NAC (CDDP + CPT-11), adjuvant (S-1 + CDDP)	None	Alive (24 months)	Nakai, 2013
3	68	Male	EGJ	ESD	ND	T1	ND	ND	ND	ND	Veits, 2013
4	68	Male	Lt	Surgery	9.5	T4	Positive	ND	ND	ND	Kadhim, 2016
5	57	Male	EGJ	Surgery	ND	T3	N3	Chemoradiation (details ND)	None	Alive (8 months)	Juanmartinena, 2017
6	64	Female	Mt	Surgery	4.0	T2	N1	Platinum and VP-16	Supraclavicular lymph nodes and liver (4 months)	Dead (8 months)	Yuan, 2017
7	62	Male	Mt	Surgery	6.0	T2	N2	Platinum and VP-16	Pleural effusion (2 months)	Dead (19 months)	Yuan, 2017
Our case	70	Male	EGJ	Surgery	2.5	T1	N0	None	None	Alive (4 months)	

*Mt* middle esophagus, *EGJ* esophagogastric junction, *Lt* lower esophagus, *Ut* upper esophagus, *ESD* endoscopic submucosal dissection, *NAC* neoadjuvant chemotherapy, *CDDP* cisplatin, *VP-16* etoposide, *ND* not described

Regarding the origin of esophageal NETs, Merkel cells and stem cells are reported candidates [22, 23]. Egashira et al. showed positive staining for CK20, a marker of Merkel cells, in 14.3% of esophageal MANECs and suggested c-kit and p53 to be potential markers of the origin of NEC cells [22]. On the other hand, it was suggested that gastric NECs arise from adenocarcinomas rather than from non-neoplastic neuroendocrine cells [24]. If the origin of the tumor of our case was Merkel cells, the tumor would not be expected to exhibit adenocarcinoma differentiation but to exhibit squamous cell carcinoma differentiation, so in the present case, the tumor might have originated from esophageal adenocarcinoma or stem cells rather than Merkel cells. It is suggested that the BE–esophageal adenocarcinoma–MANEC sequence is a potential mechanism in the pathway of MANEC carcinogenesis.

The specific and optimal treatment for MANEC in the esophagus is unknown due to the number of esophageal MANEC cases reported. According to previous reports, surgical treatment with regional lymph node dissection may be a treatment strategy for localized esophageal MANEC. Because the efficacy of lymph node dissection has not been demonstrated, further accumulation of cases is required. Adjuvant chemotherapy can be considered after resection, but there is no sufficient evidence [20]. While surgery may be the treatment of choice according to the WHO [2], multidisciplinary modalities can improve the prognosis of esophageal NEC even for resectable limited diseases [25]. The chemotherapy regimen administered for esophageal NEC is the same as that for small cell lung cancer, consisting of cisplatin, etoposide (VP-16), and irinotecan (CPT-11) [26].

Patients with mixed NEC in the esophagus (NEC plus adenocarcinoma or squamous cell carcinoma components) are more likely to be diagnosed at an earlier stage, and they showed a significantly longer survival, compared with patients with pure NEC (median survival, 28 vs. 15 months) [27]. The prognosis of high-grade MANEC was reported to depend on tumor stage and type [16].

## Conclusions

We have demonstrated a very rare case of MANEC developing in BE. BE can be the origin of esophageal MANEC as well as adenocarcinoma. The clinical and prognostic features of MANEC in BE are still unclear. Further studies are necessary to determine the optimal management strategy for esophageal or esophagogastric MANEC.

## Abbreviations

BE: Barrett’s esophagus
HPF: High-power field
MANEC: Mixed adenoneuroendocrine carcinoma
NEC: Neuroendocrine carcinoma
NET: Neuroendocrine tumor
WHO: World Health Organization
